# No-Reference Quality Assessment of Authentically Distorted Images Based on Local and Global Features

**DOI:** 10.3390/jimaging8060173

**Published:** 2022-06-19

**Authors:** Domonkos Varga

**Affiliations:** Ronin Institute, Montclair, NJ 07043, USA; domonkos.varga@ronininstitute.org

**Keywords:** no-reference image quality assessment, quality-aware features, image statistics

## Abstract

With the development of digital imaging techniques, image quality assessment methods are receiving more attention in the literature. Since distortion-free versions of camera images in many practical, everyday applications are not available, the need for effective no-reference image quality assessment algorithms is growing. Therefore, this paper introduces a novel no-reference image quality assessment algorithm for the objective evaluation of authentically distorted images. Specifically, we apply a broad spectrum of local and global feature vectors to characterize the variety of authentic distortions. Among the employed local features, the statistics of popular local feature descriptors, such as SURF, FAST, BRISK, or KAZE, are proposed for NR-IQA; other features are also introduced to boost the performances of local features. The proposed method was compared to 12 other state-of-the-art algorithms on popular and accepted benchmark datasets containing RGB images with authentic distortions (CLIVE, KonIQ-10k, and SPAQ). The introduced algorithm significantly outperforms the state-of-the-art in terms of correlation with human perceptual quality ratings.

## 1. Introduction

With the considerable advancements made in digital imaging and technology and the easy availability of cheap image-capturing devices, a large number of digital images are captured by non-technical users every day. As a consequence, people upload huge amounts of images and videos to the internet and extensively use streaming applications. In addition, visual information represents 85% of the information that is usable for human beings. Therefore, the quality assessment of digital images is of great importance and a hot research topic, along with several practical applications, such as benchmarking computer vision algorithms [1], monitoring the quality of network visual communication applications [2], fingerprint image evaluation [3], medical imaging applications [4], evaluating image compression [5], or denoising [6] algorithms. Image degradations may occur due to various reasons, such as noise, blurring, fading, or blocking artifacts. Further, the mentioned degradations can be introduced in all phases of the imaging process, such as acquisition, compression, transmission, decompression, storage, and display.

Traditionally, image quality assessment (IQA) algorithms are divided into three distinct classes in the literature with respect to the accessibility of the reference (distortion-free) images [7], i.e., no-reference (NR), full-reference (FR), and reduced-reference (RR). As the idioms indicate, NR-IQA algorithms have absolutely no access to the reference images, FR-IQA methods have full access to the reference images, while RR-IQA approaches have partial information about the reference images. In the literature, NR-IQA is recognized as a more difficult research task than the other two classes due to the complete lack of reference images [8].

In this paper, we introduce a novel NR-IQA model that relies on the fusion of local and global image features. Namely, many NR-IQA methods [9,10,11,12] characterize digital images with global features that are computed using the whole image. This approach is effective in case of artificial distortions (such as JPEG or JPEG2000 compression noise), since they are distributed uniformly in the image. However, authentically or naturally distorted images are often contaminated by noise locally. To improve the perceptual quality estimation of authentically distorted images, we combined novel local and global feature vectors using the statistics of local feature descriptors and some powerful perceptual features. Specifically, 93 features were employed, from which, 80 were introduced for NR-IQA in this study. It was empirically proved that the statistics of local feature descriptors are quality-aware features (since they interpret local image regions from the viewpoint of a human visual system); combining them with global features results in a higher performance than the current state-of-the-art. Our proposed method is code-named as *FLG-IQA*, referring to the fact that it is based on the fusion of local and global features.

The rest of this study is arranged as follows. In Section 2, related and previous papers are reviewed and outlined. Section 3 provides an overview of the used benchmark databases and evaluation metrics. Moreover, it describes the proposed approach in detail. Section 4 presents experimental results. Specifically, an ablation study is introduced to explain the effects of individual features. Moreover, a comparison to 12 other state-of-the-art methods is presented. Finally, this study is concluded in Section 5.

## 2. Literature Review

In this section, a review of the existing NR-IQA algorithms is given. For more comprehensive summaries about this topic, interested readers can refer to the studies by Zhai et al. [7], Mohammadi et al. [13], Yang et al. [14], and the book by Xu et al. [15].

NR-IQA methods can be further divided into training-free and machine learning-based classes. Our proposed method falls into the machine learning-based class; thus, this state-of-the-art study mainly focuses on this category. As the terminology indicates, machine learning-based algorithms incorporate some kind of machine learning algorithm to provide an estimation of the perceptual quality of a digital image, while training-free methods do not contain any training steps or rely on machine learning techniques using distorted images. For example, Venkatanath et al. [16] proposed the perception-based image quality evaluator (PIQE), which estimates the image qualities of distorted images using mean subtracted contrast normalized coefficients calculated at all pixel locations. In contrast, the naturalness image quality evaluator (NIQE) proposed by Mittal et al. [17] determines the distance between naturalness features extracted from the distorted image and the features obtained beforehand from distortion-free, pristine images to quantify perceptual image quality. NIQE was further developed in [18,19]. Namely, Zhang et al. [18] improved NIQE by using a Bhattacharyya-like distance [20] between the learned multivariate Gaussian model from the pristine images and those of the distorted images. In contrast, Wu et al. [19] boosted NIQE by more complex features to increase the prediction performance. Recently, Leonardi et al. [21] utilized deep features extracted from a pre-trained convolutional neural network to construct an opinion–unaware method using the correlations through the Gramian matrix between feature maps.

In the literature, various types of machine learning-based NR-IQA methods can be found. Namely, many machine learning-based algorithms rely on natural scene statistics (NSS), which is a powerful tool in characterizing image distortions. The main assumption of NSS is that digital images of high quality follow a sort of statistical regularity and distorted images significantly deviate from this pattern [22]. For instance, Saad et al. [23] examined the statistics of discrete cosine transform (DCT) coefficients. Specifically, a generalized Gaussian distribution (GGD) model was fitted on the DCT coefficients and its parameters were utilized as quality-aware features and mapped onto a perceptual quality score with the help of a support vector regressor (SVR). Another line of papers focused on the wavelet transform to extract quality-aware features. For example, Moorthy and Bovik [24] carried out a wavelet transform over three scales and three orientations; similar to [23], GGDs were fitted to the subband coefficients and their parameters were used as quality-aware features. This method was further improved in [25], where the correlations across scales, subbands, and orientations were also used as quality-aware features. In contrast, Tang et al. [26] extracted quality-aware features from complex pyramid wavelet coefficients. In [27], the authors used color statistics, such as NSS. Specifically, mean subtracted contrast normalized (MSCN) coefficients were created from the color channels of different color spaces. Next, GGD was fitted on these coefficients; similar to the previously mentioned methods, its parameters were used to map onto quality scores. Similar to [27], Mittal et al. [9] applied MSCN coefficients to characterize perceptual quality but they extracted them from the spatial domain. Other proposed models [28,29,30] utilized the statistics of local binary patterns [31] to characterize image texture degradation in the presence of noise and distortions. In [32], Freitas et al. presented a performance comparison of a wide range of local binary pattern types and variants for NR-IQA. Ye and Doerman [33,34] applied the first visual codebooks for NR-IQA. More specifically, Gabor filters were applied for feature extraction and visual codebook creation. Subsequently, the perceptual quality of an image was expressed as the weighted average of the codeword quality scores. In [35], an unsupervised feature learning framework was introduced where an unlabeled codebook was compiled from raw image patches using K−means clustering.

With the popularity of deep learning techniques and research, many deep learning-based methods were also proposed in the literature. For instance, Kang et al. [36] trained a convolutional neural network (CNN) with a convolutional layer (using both max and min pooling) and two fully-connected layers. Specifically, the proposed CNN was trained on image patches and the predicted scores of the patches were averaged to obtain the estimated quality of the whole input image. In contrast, Bianco et al. [37] introduced the DeepBIQ architecture, which extracts deep features with the help of a pre-trained CNN from multiple image patches to compile feature vectors that were mapped onto perceptual quality with a SVR. Similarly, Gao et al. [38] relied on a pre-trained CNN but multi-level feature vectors were extracted through global average pooling layers. Sheng et al. [39] utilized the fact that visual saliency is correlated with image quality [40] and trained a saliency-guided deep CNN for NR-IQA from scratch. In contrast, Liu et al. [41] introduced the learning to rank framework [42] for NR-IQA. Specifically, the authors implemented a Siamese CNN to realize this framework. Li et al. [43] implemented a novel loss function (very similar to Pearson’s linear correlation coefficient) to provide the CNN with a shorter convergence time and a better perceptual quality estimation performance. Celona and Schettini [44] trained a novel network, which processes input images at multiple scales, trained jointly by considering NR-IQA as regression, classification, and pairwise ranking, simultaneously.

## 3. Materials and Methods

### 3.1. Materials

In this subsection, the applied IQA benchmark databases are described. Further, the applied evaluation methodology and implementation environment are given.

#### 3.1.1. Applied IQA Databases

**Figure 1:**
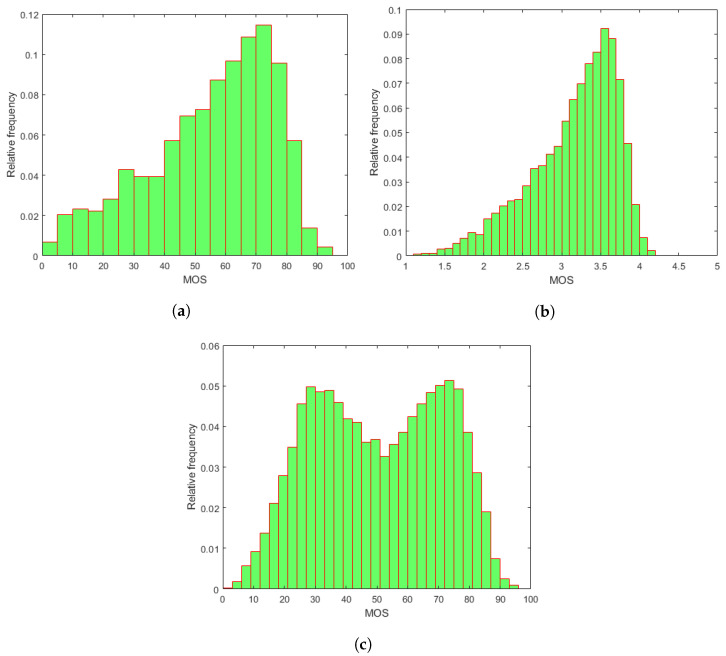
Empirical MOS distributions in the applied IQA databases: (**a**) CLIVE [45], (**b**) KonIQ-10k [46], and (**c**) SPAQ [47].

To evaluate our proposed methods and compare them against the state-of-the-art, three publicly available databases containing RGB images with authentic distortions were utilized—CLIVE [45], KonIQ-10k [46], and SPAQ [47]. The main properties of the applied IQA databases are given in Table 1. The empirical mean opinion score (MOS) distributions of the utilized IQA benchmark databases containing authentic distortions are depicted in Figure 1. In the field of IQA, MOS corresponds to the arithmetic mean of the collected individual quality ratings. Further, those distortions are considered authentic and were introduced to the images during the daily (usually non-expert) usage of imaging devices, such as overexposure, underexposure, camera jitter, motion blur, or noises from camera vibration [48].

#### 3.1.2. Evaluation

The evaluation of NR-IQA algorithms relies on the measurements of the correlations between predicted and ground truth perceptual quality scores. In the literature, Pearson’s linear correlation coefficient (PLCC), Spearman’s rank-order correlation coefficient (SROCC), and Kendall’s rank-order correlation coefficient (KROCC) are widely applied and accepted for this end [49]. As recommended by Sheikh et al. [50], non-linear mapping was carried out between the predicted and the ground truth scores before the computation of PLCC using a logistic function with five parameters,

(1) $$Q=\beta_{1}\left(\frac{1}{2}-\frac{1}{1+e^{\beta_{2}\left(Q_{p}-\beta_{3}\right)}}\right)+\beta_{4}Q_{p}+\beta_{5},$$

where $Q_{p}$ and *Q* indicate the predicted and mapped scores, respectively. Further, $\beta_{i}$, i=1,...,5 denote the fitting parameters.

As usual, in machine learning, particularly in NR-IQA, 80% of images in a database were used for training and the remaining 20% for testing. Further, median PLCC, SROCC, and KROCC values, which were measured over 100 random train–test splits, are reported in this study. PLCC, SROCC, and KROCC can be defined between vectors x and y as

(2) $$PLCC\left(x,y\right)=\frac{\sum_{i=1}^{N}\left(x_{i}-\bar{x}\right)\left(x_{i}-\bar{x}\right)}{\sqrt{\sum_{i=1}^{N}{\left(x_{i}-\bar{x}\right)}^{2}}\sqrt{\sum_{i=1}^{N}{\left(x_{i}-\bar{x}\right)}^{2}}},$$

(3) $$SROCC\left(x,y\right)=1-\frac{6\cdot \sum_{i=1}^{N}d_{i}^{2}}{N(N^{2}-1)},$$

where

(4) $$d_{i}=rank\left(x_{i}\right)-rank\left(y_{i}\right),$$

(5) $$KROCC\left(x,y\right)=\frac{N_{c}-N_{d}}{N\frac{N-1}{2}},$$

where *N* stands for the length of the vectors, $\bar{x}$ and $\bar{y}$ are the means of the vectors, $N_{c}$ and $N_{d}$ indicate the number of concordant and discordant pairs between x and y, respectively.

Our experiments were carried out in MATLAB R2021a, mainly employing the functions of the image processing and machine learning and statistics toolboxes. The main characteristics of the computer configuration applied in our experiments are outlined in Table 2.

### 3.2. Methods

**Figure 2:**
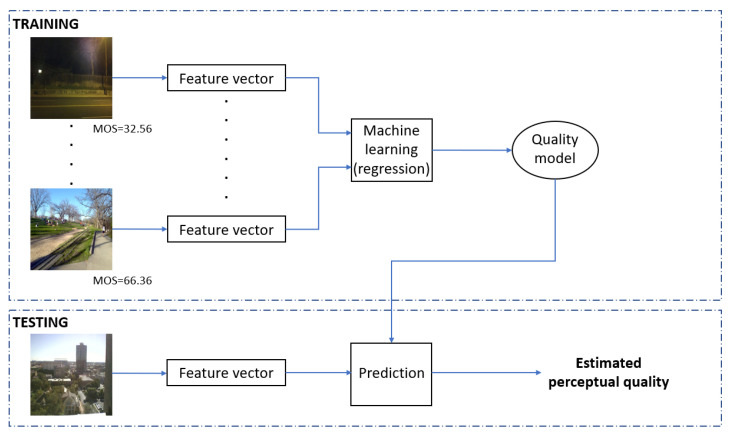
Workflow of the proposed NR-IQA algorithm.

A high-level summary of the proposed method is depicted in Figure 2, while Table 3 sums up the introduced and applied quality-aware features. As it can be seen from Figure 2, feature vectors were extracted from the set of training images to obtain a quality model with the help of a machine learning algorithm. Formally, our quality model is defined by q=G(F), where *q* is the estimated quality score, F, and *G* is a regression model that can be the Gaussian process regression (GPR) and support vector regressor (SVR). In the training phase, the model is optimized to minimize the distance between the estimated and ground truth quality scores. In the testing phase, the obtained quality model *G* was applied to estimate the perceptual quality of previously unseen test images. As already mentioned in the previous subsection, the evaluation of the quality model relies on measuring the correlation strength between the predicted and ground truth quality scores. Further, median PLCC, SROCC, and KROCC values measured over 100 random train–test splits are reported in this study.

The main (novel) contribution of the proposed method is the introduced quality-aware features outlined in Table 3. To tackle the wide variety of authentic distortions, a broad spectrum of statistical local and global features were applied. Artificial distortions (such as JPEG or JPEG2000 compression) are usually uniformly distributed in an image, which can be characterized well by global and homogeneous features [51]. However, authentic distortions often appear locally in a digital image, which can be better captured by local, non-homogeneous image features. Namely, authentic distortions are mainly introduced to the images during daily (non-expert) usage of imaging devices, such as overexposure, underexposure, camera jitter, motion blur, or noises from camera vibration [48]. In this study, the statistics of local feature descriptors supplemented by global features were introduced to construct a novel image quality model. Specifically, local feature descriptors were designed to characterize images from the human visual system’s point of view [52]. For instance, FAST [53] and Harris [54] are suitable for finding small mono-scale keypoints. On the other hand, other local feature descriptors, such as SURF [55], find multi-scale keypoints. Although they are designed to exhibit some kind of invariance against noise and illumination, perfect robustness does not exist. That is why their statistics may help characterize local image quality. Moreover, images with authentic distortions may suffer from overexposure or underexposure, which influence image content globally. The main goal of this study was to empirically prove that the fusion of local and global features can effectively estimate the perceptual quality of digital images with authentic distortions. To be specific, 93 features were applied in this study, of which, 80 were introduced for NR-IQA. The introduced features can be divided into four groups, i.e., the statistics of local feature descriptors measured on the grayscale image (f1-f35 in Table 3) and on the Prewitt-filtered image (f36-f70), Hu invariant moments computed from the binarized Sobel edge map of the input image (f66-f70), perceptual features (f78-f87), and the relative Grünwald–Letnikov derivative and gradient statistics (f88-f93). In the following subsection, each group of the features is introduced in detail.

**Table 3:** Summary of the applied features. Quality-aware features proposed by this paper are in bold.

Feature Number	Input	Feature	Number of Features
**f1-f5**	SURF [55], Grayscale image	mean, median, std, skewness, kurtosis	5
**f6-f10**	FAST [53], Grayscale image	mean, median, std, skewness, kurtosis	5
**f11-f15**	BRISK [56], Grayscale image	mean, median, std, skewness, kurtosis	5
**f16-f20**	KAZE [57], Grayscale image	mean, median, std, skewness, kurtosis	5
**f21-f25**	ORB [58], Grayscale image	mean, median, std, skewness, kurtosis	5
**f26-f30**	Harris [54], Grayscale image	mean, median, std, skewness, kurtosis	5
**f31-f35**	Minimum Eigenvalue [59], Grayscale image	mean, median, std, skewness, kurtosis	5
**f36-f40**	SURF [55], Filtered image	mean, median, std, skewness, kurtosis	5
**f41-f45**	FAST [53], Filtered image	mean, median, std, skewness, kurtosis	5
**f46-f50**	BRISK [56], Filtered image	mean, median, std, skewness, kurtosis	5
**f51-f55**	KAZE [57], Filtered image	mean, median, std, skewness, kurtosis	5
**f56-f60**	ORB [58], Filtered image	mean, median, std, skewness, kurtosis	5
**f61-f65**	Harris [54], Filtered image	mean, median, std, skewness, kurtosis	5
**f66-f70**	Minimum Eigenvalue [59], Filtered image	mean, median, std, skewness, kurtosis	5
**f71-f77**	Binary image	Hu invariant moments [60]	7
f78-f87	RGB image	Perceptual features	10
**f88**	GL-GM map (α=0.3)	histogram variance	1
**f89**	GL-GM map (α=0.6)	histogram variance	1
**f90**	GL-GM map (α=0.9)	histogram variance	1
f91	GM map [61]	histogram variance	1
f92	RO map [61]	histogram variance	1
f93	RM map [61]	histogram variance	1

### 3.3. Statistics of Local Feature Descriptors

In contrast to artificially distorted images, where distortions are uniformly distributed, natural or authentic distortions are often present locally in images. This is why the statistics of local feature descriptors are proposed for quality-aware features in this study. In this study, we calculated the statistics of the following local feature detectors: SURF [55], FAST [53], BRISK [56], KAZE [57], ORB [58], Harris [54], and minimum eigenvalue [59]. Specifically, the strongest 250 interest points were detected separately with each of the above-mentioned local feature detectors and the adherent feature vectors were determined. In this study, the following statistics of the obtained feature vectors were considered as local features: mean, median, standard deviation, skewness, and kurtosis. The skewness of a vector x containing *n* elements is calculated as

(6) $$s=\frac{\frac{1}{n}\sum_{i=1}^{n}{\left(x_{i}-\bar{x}\right)}^{3}}{{\left(\sqrt{\frac{1}{n}\sum_{i=1}^{n}{\left(x_{i}-\bar{x}\right)}^{2}}\right)}^{2}},$$

while its kurtosis can be given as

(7) $$k=\frac{\frac{1}{n}\sum_{i=1}^{n}{\left(x_{i}-\bar{x}\right)}^{4}}{{\left(\frac{1}{n}\sum_{i=1}^{n}{\left(x_{i}-\bar{x}\right)}^{2}\right)}^{3}},$$

where $\bar{x}$ stands for the arithmetic mean of x. Specifically, the statistics of the features around the feature points were calculated and their arithmetic means were considered as quality-aware features. Moreover to increase the distinctiveness of local feature detectors [62], the statistics of the local feature detectors were also extracted from the filtered version of the input image obtained by Prewitt operators [63].

### 3.4. Hu Invariant Moments

The moment (m) is a projection of a function (in image processing an image I(x,y)) to the polynomial basis. Formally, it can be written as

(8) $$m_{i,j}=\sum_{x=1}^{N}\sum_{y=1}^{M}x^{i}y^{j}I\left(x,y\right),$$

where *N* and *M* are the width and the height of image I(x,y), respectively. The order (r) of the moment is defined as r=i+j. It can be easily pointed out that $m_{0,0}$ is the mass of the image. Further, $m_{1,0}/m_{0,0}$ and $m_{0,1}/m_{0,0}$ are the coordinates of the center of gravity of the image. The central moment (μ) is defined as

(9) $$\mu_{i,j}=\sum_{x=1}^{N}\sum_{y=1}^{M}{\left(x-\frac{m_{1,0}}{m_{0,0}}\right)}^{i}{\left(y-\frac{m_{0,1}}{m_{0,0}}\right)}^{j}I\left(x,y\right).$$

It is evident that $\mu_{0,0}=m_{0,0}$. Next, the normalized central moment (η) is given as

(10) $$\eta_{i,j}=\frac{\mu_{i,j}}{{\left(\mu_{0,0}\right)}^{\lambda }},$$

where

(11) $$\lambda =\frac{i+j}{2}+1.$$

Hu [60] proposed seven invariant moments, which were defined using the normalized central moment, such as

(12) $$\phi_{1}=\eta_{2,0}+\eta_{0,2},$$

(13) $$\phi_{2}={\left(\eta_{2,0}+\eta_{0,2}\right)}^{2}+4{\left(\eta_{1,1}\right)}^{2},$$

(14) $$\phi_{3}={\left(\eta_{3,0}-3\eta_{1,2}\right)}^{2}+{\left(3\eta_{2,1}-\eta_{0,3}\right)}^{2},$$

(15) $$\phi_{4}={\left(\eta_{3,0}+\eta_{1,2}\right)}^{2}+{\left(\eta_{2,1}+\eta_{0,3}\right)}^{2},$$

(16) $$\begin{array}{c} \phi_{5}=\left(\eta_{3,0}-3\eta_{1,2}\right)\left(\eta_{3,0}+\eta_{1,2}\right)\left[{\left(\eta_{3,0}+\eta_{1,2}\right)}^{2}-3{\left(\eta_{2,1}+\eta_{0,3}\right)}^{2}\right]+\\ \;\;\;\;\;\;\;\;\;\;\left(3\eta_{2,1}-\eta_{0,3}\right)\left(\eta_{2,1}+\eta_{0,3}\right)\left[3{\left(\eta_{3,0}+\eta_{1,2}\right)}^{2}-{\left(\eta_{2,1}+\eta_{0,3}\right)}^{2}\right] \end{array}$$

(17) $$\phi_{6}=\left(\eta_{2,0}-\eta_{0,2}\right)\left[{\left(\eta_{3,0}+\eta_{1,2}\right)}^{2}-{\left(\eta_{2,1}+\eta_{0,3}\right)}^{2}\right]+4\eta_{1,1}\left(\eta_{3,0}+\eta_{1,2}\right)\left(\eta_{2,1}+\eta_{0,3}\right)$$

(18) $$\begin{array}{c} \phi_{7}=\left(3\eta_{2,1}-\eta_{0,3}\right)\left(\eta_{3,0}+\eta_{1,2}\right)\left[{\left(\eta_{3,0}+\eta_{1,2}\right)}^{2}-3{\left(\eta_{2,1}+\eta_{0,3}\right)}^{2}\right]-\\ \;\;\;\;\;\;\;\;\;\;\left(\eta_{3,0}-3\eta_{1,2}\right)\left(\eta_{2,1}+\eta_{0,3}\right)\left[3{\left(\eta_{3,0}+\eta_{1,2}\right)}^{2}-{\left(\eta_{2,1}+\eta_{0,3}\right)}^{2}\right]. \end{array}$$

The human visual system is very sensitive to edge and contour information, since this information gives reliable implications about the structure of an image [64,65]. To characterize the contour information of an image, the Hu invariant moments [60] of the image’s binary Sobel edge map were used as quality-aware features. The horizontal and vertical derivative approximations of input image I were determined as

(19) $$G_{x}=\left(\begin{array}{ccc} +1 & 0 & -1\\ +2 & 0 & -2\\ +1 & 0 & -1 \end{array}\right)*I,$$

(20) $$G_{y}=\left(\begin{array}{ccc} +1 & +2 & +1\\ 0 & 0 & 0\\ -1 & -2 & -1 \end{array}\right)*I,$$

where ∗ is the convolution operator. Next, the gradient magnitude was computed as

(21) $$G=\sqrt{G_{x}^{2}+G_{y}^{2}}.$$

The binary Sobel edge map was obtained from the gradient magnitude by applying a cutoff threshold corresponding to the quadruple of G’s mean.

### 3.5. Perceptual Features

Some perceptual features, which are proved to be consistent with human quality judgments [66], were also built into our model. In the following, an RGB color image is denoted by *I* and $I^{c}\left(c\in \left(R,G,B\right)\right)$ is a color channel of input image *I*. Moreover, *x* stands for the pixel coordinate and we assume that *I* has *N* pixels.

1. Blur: It is probably the most dominant source of perceptual image quality deterioration in digital imaging [67]. To quantify the emergence of the blur effect, the blur metric of Crété-Roffet et al. [68], which is based on the measurements of intensity variations between neighboring pixels, was implemented due to its low computational costs.
2. Colorfulness: In [69], Choi et al. pointed out that colorfulness is a critical component in human image quality judgment. We determined colorfulness using the following formula proposed by Hasler and Suesstrunk [70]:
  (22) $$CF=\sqrt{\sigma_{rg}^{2}+\sigma_{yb}^{2}}+\frac{3}{10}\sqrt{\mu_{rg}^{2}+\mu_{yb}^{2}},$$
  where σ and μ stand for the standard deviation and the mean of the matrices denoted in the subscripts, respectively. Specifically, these matrices are given as:
  (23) rg=R−G,
  (24) $$yb=\frac{1}{2}\left(R+G\right)-B,$$
  where *R*, *G*, and *B* are the red, green, and blue color channels, respectively.
3. Chroma: It is one of the relevant image features among a series of color metrics in the CIELAB color space. Moreover, chroma is significantly correlated with haze, blur, or motion blur in the image [70]. It is defined as
  (25) $$Chroma\left(x\right)=\sqrt{{\left(a(x)\right)}^{2}+{\left(b(x)\right)}^{2}},$$
  where *a* and *b* are the corresponding color channels of the CIELAB color space. The arithmetic mean of Chroma(x) was used as a perceptual feature in our model.
4. Color gradient: The estimated color gradient magnitude (CGM) map is defined as
  (26) $$CGM\left(x\right)=\sum_{c\in (R,G,B)}\sqrt{{\left(I_{x}^{c}\left(x\right)\right)}^{2}+{\left(I_{y}^{c}\left(x\right)\right)}^{2}},$$
  where $I_{x}\left(x\right)$ and $I_{y}\left(x\right)$ stand for the approximate directional derivatives in the horizontal *x* and vertical *y* directions of I(x), respectively. In our study, the mean and standard deviations of CGM(x) are utilized as quality-aware features.
5. Dark channel feature (DCF): In the literature, Tang et al. [71] proposed DCF [72] for image quality assessment, since it can effectively identify haze effects in images. A dark channel is defined as
  (27) $$I^{dark}\left(x\right)=\min_{y\in \Omega (x)}\left(\min_{c\in (R,G,B)}I^{c}\left(y\right)\right),$$
  where Ω(x) denotes the image patches around the pixel location *x*. In our implementation, an image patch corresponds to a 15×15 square. Next, the DCF is defined as
  (28) $$DCF=\frac{1}{\left||S|\right|}\sum_{i\in S}\frac{I^{dark}\left(i\right)}{\sum_{c\in (R,G,B)}I^{c}\left(i\right)},$$
  where ||S|| is the size of image *I*.
6. Michelson contrast: Contrast is one of the most fundamental characteristics of an image, since it influences the ability to distinguish objects from each other in an image [73]. Thus, contrast information is built into our NR-IQA model. The Michelson contrast measures the difference between the maximum and minimum values of an image [74], defined as
  (29) $$C_{Michelson}=\sum_{c\in (R,G,B)}\frac{\max\left(I^{c}\left(x\right)\right)-\min\left(I^{c}\left(x\right)\right)}{\max\left(I^{c}\left(x\right)\right)+\min\left(I^{c}\left(x\right)\right)}.$$
7. Root mean square (RMS) contrast is defined as
  (30) $$C_{RMS}=\sqrt{\frac{1}{N}\sum_{x=0}^{N-1}{\left(I\left(x\right)-\bar{I}\right)}^{2}}$$
  where $\bar{I}$ denotes the mean luminance of I(x).
8. Global contrast factor (GCF): Contrary to Michelson and RMS contrasts, GCF considers multiple resolution levels of an image to estimate human contrast perception [75]. It is defined as
  (31) $$GCF=\sum_{i=1}^{9}w_{i}C_{i},$$
  where $C_{i}$’s are the average local contrasts and $w_{i}$’s are the weighting factors. The authors examined nine different resolution levels, which is why the number of weighting factors are nine; $w_{i}$’s are defined as
  (32) $$w_{i}=\left(-0.406385\cdot \frac{i}{9}+0.334573\right)\cdot \frac{i}{9}+0.0877526,$$
  which is a result of an optimum approximation from the best fitting [75]. The local contrasts are defined as follows. First, the image of size w×h is rearranged into a one-dimensional vector using row-wise sorting. Next, the local contrast $l_{C_{i}}$ in pixel location *i* is defined as
  (33) $$l_{C_{i}}=\frac{|L_{i}-L_{i-1}\left|+\right|L_{i}-L_{i+1}\left|+\right|L_{i}-L_{i-w}\left|+\right|L_{i}-L_{i+w}|}{4},$$
  where $L_{i}$ denotes the pixel value at location *i* after gamma correction (γ=2.2). Finally, the average local contrast at resolution *i* (denoted by $C_{i}$ in Equation (31)) is determined as the average of all $l_{C_{i}}$’s over the entire image.
9. Entropy: It is a quantitative measure of the image’s carried information [76]. Typically, an image with better quality is able to transmit more information. This is why entropy was chosen as a quality-aware feature. The entropy of a grayscale image is defined as
  (34) $$E=-\sum_{n=0}^{255}p\left(n\right)\cdot \log_{2}\left(p(n)\right),$$
  where p(·) consists of the normalized histogram counts of the grayscale image.

Table 4 illustrates the average values of the perceptual features in five equal MOS intervals of CLIVE [45] from very low image quality to very high image quality. From these numerical results, it can be observed that the mean values of the applied perceptual features are roughly proportional with the perceptual quality class. For instance, the mean values of several perceptual features (the mean and the standard deviations of the color gradient, Michelson contrast, RMS contrast, GCF, and entropy) monotonically increase with the quality class. Similarly, Table 5 illustrates the standard deviation values of the perceptual features in five equal MOS intervals of CLIVE [45] from very low image quality to very high image quality. It can be seen that the standard deviation values are also roughly proportional to the perceptual quality classes. For instance, the standard deviation values of several perceptual features (color gradient-mean, DCF, Michelson contrast, RMS contrast, and entropy) exhibit a remarkable proportionality with the perceptual quality classes.

### 3.6. Relative Grünwald–Letnikov Derivative and Gradient Statistics

In image processing, image gradient is one of the most widely used features [77,78,79,80] and a strong predictive factor for image quality [61,81]. To characterize gradient degradation in the presence of image noise, the idea of gradient magnitude (GM), relative gradient orientation (RO), and relative gradient magnitude (RM) maps were applied from [61], on the one hand. On the other hand, the idea of GM maps was generalized and developed further using the Grünwald–Letnikov (GL) derivative [82]. Once, GM, RO, and RM maps were computed—following the recommendations of [61]—their histogram variances were used as quality-aware features. Given a normalized histogram h(x), the histogram variance is defined as

(35) $$Var\left(h\left(x\right)\right)=\sum_{i=1}^{n}\left(h\left(x\right)-\bar{h}\right),$$

where *n* is the length of h(x) and $\bar{h}$ is the mean of h(x).

The Grünwald–Letnikov derivative, introduced by Anton Karl Grünwald and Aleksey Vasilievich Letnikov, enables differentiating a function a non-integer amount of times [83]. Generally, a one-dimensional function f(x) can be differentiated for any $n\in N^{+}$ using the following formula

(36) dnf(x)dxn=limh→0∑j=0n(−1)jnjf(x−jh)h2,

where

(37) nj=n(n−1)...(n−j+1)j!.

Grünwald and Letnikov invented an approach, which enables taking the derivative of a function by arbitrary, non-integer α-times. Formally, it has been written

(38) Dx0,xGLαf(x)=limh→01hα∑j=0[x−x0h](−1)jΓ(α+1)Γ(j+1)Γ(α−j+1)f(x−jh),

where Γ(·) is the Gamma-function, Dx0,xGLαf(x) is the αth order Grünwald–Letnikov derivative, and *x* and $x_{0}$ stand for the upper and lower bounds, respectively. For a discrete two-dimensional signal (in the context of image processing: a digital image), I(x,y), the GL derivative in the *x*-direction can be defined as

(39) DGLαIx(x,y)=I(x,y)−αI(x−1,y)+α(α−1)2I(x−2,y).

Similarly, in the *y*-direction

(40) DGLαIy(x,y)=I(x,y)−αI(x,y−1)+α(α−1)2I(x,y−2).

Similar to the traditional definition, the GL derivate can be given as

(41) DGLαI(x,y)=(DGLαIx(x,y))2+(DGLαIy(x,y))2.

Although the physical meaning of the GL fractional derivative is not absolutely understandable, it is important to notice that GL derivatives, in contrast to integer derivatives, do not have local characters [84]. Namely, their derivatives depend on the entire functions. In previous work [85], the combination of global and local variations of an image using GL derivatives (global) and image gradients (local) proved to be beneficial for FR-IQA. Note that the full-reference (FR) setting aims to evaluate an image using reference images without distortions. In this study, in order to characterize global variations of images for no reference (NR)-IQA, the computation of the GM [61] map was modified by using the equations of the GL derivative, as in Equations (39)–(41). More specifically, three GL-GM maps were computed with α=0.3, α=0.6, and α=0.9, respectively, and their histogram variances were taken as quality-aware features.

The GM map can be given very similarly to Equation (41):

(42) $$GM\left(x,y\right)=\sqrt{{\left(I_{x}\left(x\right)\right)}^{2}+{\left(I_{y}\left(x\right)\right)}^{2}}$$

where $I_{x}\left(x\right)$ and $I_{y}\left(x\right)$ stand for the approximate directional derivatives in the horizontal *x* and vertical *y* directions of I(x), respectively.

The definition of the RO map is as follows [61]. First, the gradient orientation needs to be defined:

(43) $$\theta \left(x,y\right)=\arctan\left(\frac{I_{y}\left(x,y\right)}{I_{x}\left(x,y\right)}\right).$$

The RO map can be given as

(44) $$RO\left(x,y\right)=\theta \left(x,y\right)-\theta_{AVE}\left(x,y\right)$$

where $\theta_{AVE}\left(x,y\right)$ is the local average orientation; the authors define as

(45) $$\theta_{AVE}\left(x,y\right)=\arctan\left(\frac{I_{y,AVE}\left(x,y\right)}{I_{x,AVE}\left(x,y\right)}\right),$$

where the average directional derivatives are defined as

(46) $$I_{y,AVE}\left(x,y\right)=\frac{1}{M\cdot N}\sum \sum_{(m,n)\in W}I_{y}\left(x-m,y-n\right),$$

and

(47) $$I_{x,AVE}\left(x,y\right)=\frac{1}{M\cdot N}\sum \sum_{(m,n)\in W}I_{x}\left(x-m,y-n\right),$$

where *W* stands for the local neighborhood over the values that are computed. Similar to the RO map, the RM map can be given as

(48) $$RM\left(x,y\right)=\sqrt{{\left(I_{x}\left(x,y\right)-I_{x,AVE}\left(x,y\right)\right)}^{2}+{\left(I_{y}\left(x,y\right)-I_{y,AVE}\left(x,y\right)\right)}^{2}}.$$

## 4. Results

In this section, our results are presented. Specifically, Section 4.1 consists of an ablation study to analyze the performance of the proposed, individual quality-aware features. Next, Section 4.2 describes the results of a performance comparison to other state-of-the-art NR-IQA methods.

### 4.1. Ablation Study

**Figure 3:**
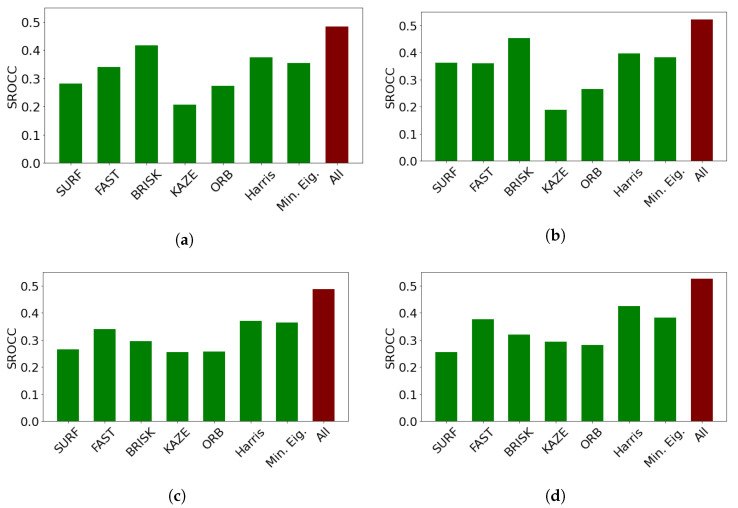
Comparison of the statistics of local feature descriptors as quality-aware features in CLIVE [45]. Median SROCC values were measured over 100 random train–test splits. (**a**) RGB image, SVR, (**b**) RGB image, GPR, (**c**) filtered image, SVR, (**d**) filtered image, GPR.

In this subsection, an ablation study on CLIVE [45] is presented to reason the design choices and demonstrate the individual performances of the proposed quality-aware features. The results are summarized in Table 6. From these results, it can be observed that the statistics of feature descriptors are quality-aware features and can deliver a rather strong performance. However, the set of the applied perceptual features delivers the strongest performance. It can also be seen that combining the statistics of local feature descriptors with global features results in improved performance. Moreover, GPR with the rational quadratic kernel function outperforms SVR with the Gaussian kernel function as a regressor for all of the proposed quality-aware features. Figure 3 provides detailed results for all local feature descriptors. On their own, all local feature descriptors can provide weak or mediocre performances. However, their concatenations provide rather strong performances. We attribute this result to the ability of local feature descriptors to diversely characterize local image distortions. Based on the above observations, we used GPR with the rational quadratic kernel function as a regressor in the proposed method, which is code-named *FLG-IQA*, referring to the fact that it is based on the fusion of local and global features.

**Figure 4:**
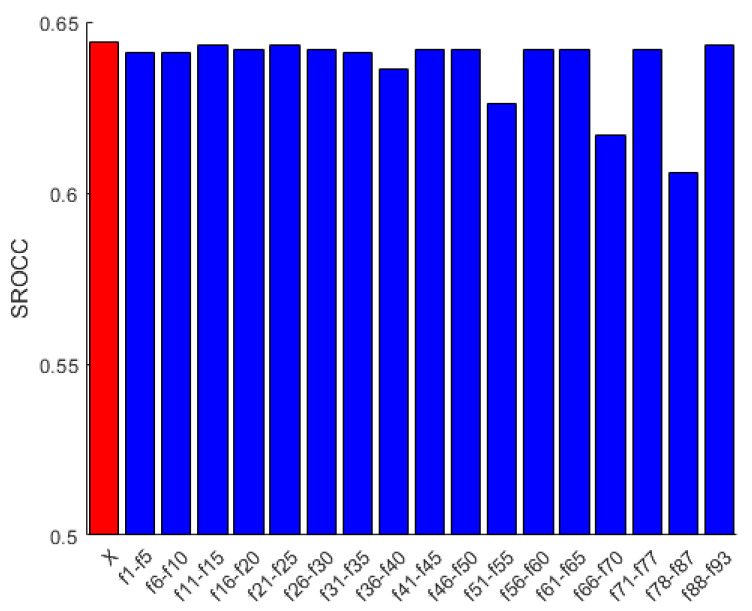
Performance in terms of SROCC of the proposed *FLG-IQA* in cases when a given feature is removed from the proposed feature vector. The performance of the entire feature vector is indicated by ‘X’. Median SROCC values were measured on CLIVE [45] after 100 random train–test splits.

To demonstrate that all features are relevant in *FLG-IQA*, we present an experiment based on the evaluation protocol described in Section 3.1.2 and using the CLIVE [45] database, in which a given feature is eliminated from the entire feature vector. As demonstrated in Figure 4, all features are important. If one of the applied features is removed, the performance of *FLG-IQA* falls back. However, the statistics of SURF, KAZE, and minimum eigenvalue local feature descriptors on the Prewitt-filtered image are the most decisive to the performance of *FLG-IQA* from the statistics of local feature descriptors. Moreover, perceptual features have the most contributing effects to the performance of the proposed method. If we contrast the results in Figure 4 with results in Figure 3d and Table 6, the following interesting fact can be observed. Features that have strong effects on the performance of *FLG-IQA*, do not have always superior performance individually. For instance, if the statistics of the minimum eigenvalue local feature descriptor in the Prewitt-filtered image are removed, a significant performance drop can be observed. On the other hand, its individual performance lags behind those of the Harris statistics on the filtered image, although Harris has a rather strong individual performance. This indicates that the statistics of local feature descriptors are quality-aware feature vectors and complement each other in NR-IQA. Further, perceptual features have strong individual performances and their removal from the feature vector throw back the performance, indicating that they are very strong predictors of the perceptual image quality.

**Figure 5:**
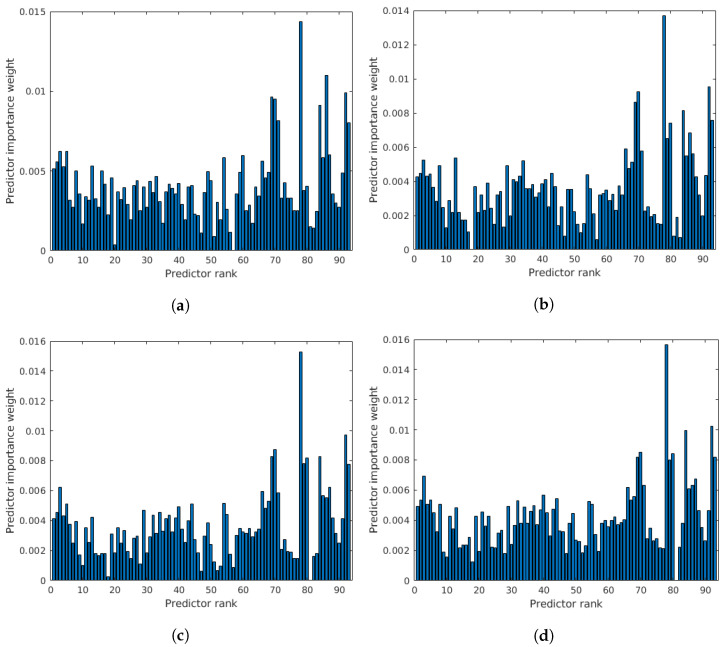
Results of the RReliefF algorithm on the features extracted from the images of CLIVE [45]. (**a**) k=1 nearest neighbours, (**b**) k=3 nearest neighbours, (**c**) k=5 nearest neighbours, (**d**) k=7 nearest neighbours.

To further prove that all entries of the proposed feature vector are important, the rank importance of all predictors was investigated using the RReliefF algorithm [86]. Namely, the main idea behind RReliefF [87] is to estimate the discriminative power of features based on their ability on how well they differentiate between instances that lie near each other in the feature space. To this end, RReliefF penalizes those predictors that provide different values to neighbors with the same response values. On the other hand, it rewards those predictors that give different values to neighbors with different response values. Further, the number of examined neighbors is an input parameter of RReliefF. For all details about RReliefF, we refer to the paper by Robnik-Sikonja and Kononenko [88]. The results of the RReliefF algorithm—using 1,3,5, and 7 nearest neighbors—on the features extracted from the images of CLIVE [45], are depicted in Figure 5. From these results, it can be seen that all entries of the proposed feature vector are important since the weights of importance are non-negative in all cases.

### 4.2. Comparison to the State-of-the-Art

**Figure 6:**
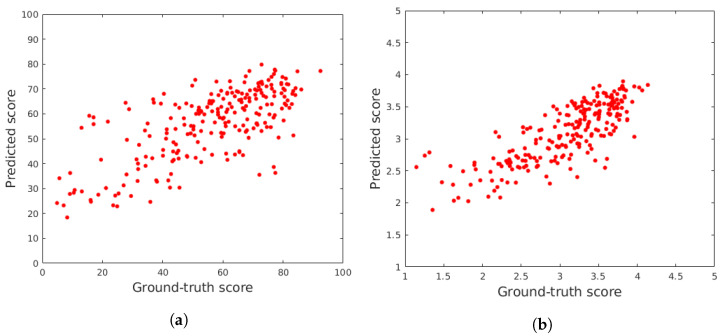
Ground truth scores versus predicted scores in (**a**) CLIVE [45] and (**b**) KonIQ-10k [46] test sets.

In this subsection, the proposed *FLG-IQA* algorithm is compared to several state-of-the-art methods, such as BLIINDS-II [23], BMPRI [12], BRISQUE [9], CurveletQA [10], DIIVINE [25], ENIQA [89], GRAD-LOG-CP [11], GWH-GLBP [90], NBIQA [91], OG-IQA [61], PIQE [16], and SSEQ [92], whose original MATLAB source codes can be found online. The above-mentioned algorithms were evaluated in the same environment with the same evaluation protocol as ours. Since PIQE [16] is a training-free method without any machine learning technique, it was directly evaluated in the full datasets. The results are summarized in Table 7 and Table 8, where it can be seen that the proposed *FLG-IQA* is able to outperform all other considered methods in three large IQA databases with authentic distortions, i.e., CLIVE [45], KonIQ-10k [46], and SPAQ [47]. Table 9 illustrates the direct and weighted average performance values obtained from the achieved results on the used IQA benchmark databases. It can be observed that the proposed *FLG-IQA* outperforms all other examined state-of-the-art algorithms by a large margin in this comparison. Specifically, *FLG-IQA* surpasses the second best methods by approximately 0.05 in terms of PLCC, SROCC, and KROCC in direct and weighted averages as well. In general, all methods achieved higher values in the case of the weighted average, which implies that the examined methods tend to perform better on larger databases. As an illustration of the results, Figure 6 depicts ground truth versus predicted scores in CLIVE [45] and KonIQ-10k [46] test sets, respectively.

To prove that the achieved performance difference against the state-of-the-art in CLIVE [45], KonIQ-10k [46], and SPAQ [47] was significant, significance tests were also carried out. Specifically, one-sided *t*-tests were applied between the 100 SROCC values provided by the proposed FLG-IQA method and one other examined state-of-the-art algorithm. Further, the null hypothesis was that the mean SROCC values of the two sets were equal to each other at a confidence level of 95%. The results of the significance tests are summarized in Table 10 where symbol 1(−1) denotes that the proposed FLG-IQA is significantly better (worse) than the algorithm represented in the row of the table on the IQA benchmark database represented in the column. From the presented results, it can be seen that FLG-IQA is significantly better than the state-of-the-art in the utilized IQA benchmark databases containing authentic distortions.

**Figure 7:**
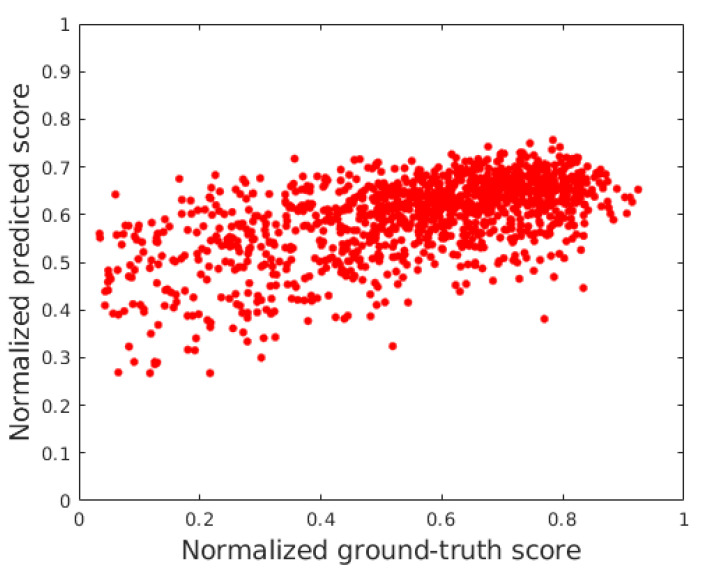
Normalized ground truth scores versus normalized predicted score scatter plot of FLG-IQA in the cross-database test.

The effectiveness of the proposed FLG-IQA was further proved in a cross-database test where the examined state-of-the-art algorithm and the proposed method were trained on the large KonIQ-10k [46] and tested in CLIVE [45]. The results of this test are summarized in Table 11. From the presented numerical results, it can be seen that the proposed method provides a significantly higher performance than the other methods. Specifically, FLG-IQA performs better than the second-best method bu approximately 0.11 in terms of PLCC and 0.07 in terms of SROCC, respectively. Figure 7 depicts the results of FLG-IQA in the cross database in normalized ground truth scores versus a normalized predicted score scatter plot.

## 5. Conclusions

In this paper, a novel NR-IQA method for authentically distorted images was introduced. Specifically, a diverse set of local and global quality-aware features was proposed and applied with a GPR with the rational quadratic kernel function to obtain a perceptual quality estimator. The main idea behind the usage of local feature descriptor statistics was that these feature descriptors interpret local image regions from the human visual system’s viewpoint. The features were studied by taking into consideration their effects on the performance of perceptual quality estimation. The numerical comparison to 12 other state-of-the-art methods on three popular benchmark databases (CLIVE [45], KonIQ-10k [46], and SPAQ [47]) proved the superior performance of the proposed method. Our future work will involve boosting the quality-aware properties of the local feature descriptors by applying bio-inspired filters. 

## Figures and Tables

**Table 1:** Summary about the applied benchmark IQA databases with authentic distortions. DSLR: digital single-lens reflex camera. DSC: digital still camera. SPHN: smartphone.

Attribute	CLIVE [45]	KonIQ-10k [46]	SPAQ [47]
#Images	1162	10,073	11,125
Resolution	500×500	1024×768	∼4000×4000
#Subjects	8100	1,467	600
#Annotations	1400	1,200,000	186,400
Scale of quality scores	0–100	1–5	0–100
Subjective methodology	Crowdsourcing	Crowdsourcing	Laboratory
Types of cameras	DSLR/DSC/SPHN	DSLR/DSC/SPHN	SPHN
Year of publication	2017	2018	2020

**Table 2:** The applied computer configuration of the experiments.

Computer model	STRIX Z270H Gaming
Operating system	Windows 10
CPU	Intel(R) Core(TM) i7-7700K CPU 4.20 GHz (8 cores)
Memory	15 GB
GPU	Nvidia GeForce GTX 1080

**Table 4:** Mean values of perceptual features in CLIVE [45] with respect to five equal MOS intervals.

	0≤MOS<20	20≤MOS<40	40≤MOS<60	60≤MOS<80	80≤MOS≤100
Blur	0.412	0.362	0.315	0.285	0.329
Colorfulness	0.046	0.038	0.042	0.045	0.072
Chroma	15.510	13.681	14.995	15.409	21.977
Color gradient-mean	92.801	116.884	154.651	189.795	196.287
Color gradient-std	132.693	163.876	207.837	244.420	235.855
DCF	0.217	0.211	0.197	0.220	0.192
Michelson contrast	2.804	2.832	2.911	2.937	2.953
RMS contrast	0.201	0.201	0.219	0.222	0.223
GCF	5.304	5.488	6.602	6.264	6.796
Entropy	6.832	6.985	7.182	7.413	7.583

**Table 5:** Standard deviation values of perceptual features in CLIVE [45] with respect to five equal MOS intervals.

	0≤MOS<20	20≤MOS<40	40≤MOS<60	60≤MOS<80	80≤MOS≤100
Blur	0.109	0.096	0.075	0.067	0.093
Colorfulness	0.050	0.033	0.037	0.039	0.049
Chroma	12.698	8.143	9.680	8.927	11.720
Color gradient-mean	45.480	66.164	89.762	96.283	99.800
Color gradient-std	58.236	71.187	82.104	84.179	78.250
DCF	0.141	0.122	0.117	0.115	0.105
Michelson contrast	0.328	0.252	0.173	0.143	0.140
RMS contrast	0.080	0.068	0.065	0.056	0.051
GCF	1.934	1.665	1.761	1.857	1.746
Entropy	1.019	0.966	0.748	0.532	0.227

**Table 6:** Ablation study on CLIVE [45] database. Median PLCC, SROCC, and KROCC values were measured over 100 random train–test splits.

	SVR			GPR		
**Method**	**PLCC**	**SROCC**	**KROCC**	**PLCC**	**SROCC**	**KROCC**
Feature descriptors, RGB image	0.518	0.484	0.337	0.578	0.523	0.364
Feature descriptors, filtered image	0.529	0.488	0.338	0.582	0.527	0.364
Hu invariant moments	0.302	0.295	0.199	0.328	0.320	0.219
Perceptual features	0.607	0.588	0.420	0.626	0.598	0.425
GL and gradient statistics	0.528	0.492	0.343	0.541	0.495	0.343
All	0.636	0.604	0.428	0.685	0.644	0.466

**Table 7:** Comparison to the state-of-the-art in CLIVE [45] and KonIQ-10k [46] databases. Median PLCC, SROCC, and KROCC values were measured over 100 random train–test splits. The best results are in bold and the second-best results are underlined.

	CLIVE [45]			KonIQ-10k [46]		
**Method**	**PLCC**	**SROCC**	**KROCC**	**PLCC**	**SROCC**	**KROCC**
BLIINDS-II [23]	0.473	0.442	0.291	0.574	0.575	0.414
BMPRI [12]	0.541	0.487	0.333	0.637	0.619	0.421
BRISQUE [9]	0.524	0.497	0.345	0.707	0.677	0.494
CurveletQA [10]	0.636	0.621	0.421	0.730	0.718	0.495
DIIVINE [25]	0.617	0.580	0.405	0.709	0.693	0.471
ENIQA [89]	0.596	0.564	0.376	0.761	0.745	0.544
GRAD-LOG-CP [11]	0.607	0.604	0.383	0.705	0.696	0.501
GWH-GLBP [90]	0.584	0.559	0.395	0.723	0.698	0.507
NBIQA [91]	0.629	0.604	0.427	0.771	0.749	0.515
OG-IQA [61]	0.545	0.505	0.364	0.652	0.635	0.447
PIQE [16]	0.172	0.108	0.081	0.208	0.246	0.172
SSEQ [92]	0.487	0.436	0.309	0.589	0.572	0.423
FLG-IQA	**0.685**	**0.644**	**0.466**	**0.806**	**0.771**	**0.578**

**Table 8:** Comparison to the state-of-the-art in the SPAQ [47] database. Median PLCC, SROCC, and KROCC values were measured over 100 random train–test splits. The best results are in bold and the second-best results are underlined.

Method	PLCC	SROCC	KROCC
BLIINDS-II [23]	0.676	0.675	0.486
BMPRI [12]	0.739	0.734	0.506
BRISQUE [9]	0.726	0.720	0.518
CurveletQA [10]	0.793	0.774	0.503
DIIVINE [25]	0.774	0.756	0.514
ENIQA [89]	0.813	0.804	0.603
GRAD-LOG-CP [11]	0.786	0.782	0.572
GWH-GLBP [90]	0.801	0.796	0.542
NBIQA [91]	0.802	0.793	0.539
OG-IQA [61]	0.726	0.724	0.594
PIQE [16]	0.211	0.156	0.091
SSEQ [92]	0.745	0.742	0.549
FLG-IQA	**0.850**	**0.845**	**0.640**

**Table 9:** Comparison to the state-of-the-art. Direct and weighted average PLCC, SROCC, and KROCC are reported based on the results measured in CLIVE [45], KonIQ-10k [46], and SPAQ [47]. The best results are in bold and the second-best results are underlined.

	Direct Average			Weighted Average		
**Method**	**PLCC**	**SROCC**	**KROCC**	**PLCC**	**SROCC**	**KROCC**
BLIINDS-II [23]	0.574	0.564	0.397	0.620	0.618	0.443
BMPRI [12]	0.639	0.613	0.420	0.683	0.669	0.459
BRISQUE [9]	0.652	0.631	0.452	0.707	0.689	0.498
CurveletQA [10]	0.720	0.704	0.473	0.756	0.741	0.495
DIIVINE [25]	0.700	0.676	0.463	0.737	0.718	0.489
ENIQA [89]	0.723	0.704	0.508	0.778	0.765	0.565
GRAD-LOG-CP [11]	0.699	0.694	0.485	0.740	0.734	0.530
GWH-GLBP [90]	0.703	0.684	0.481	0.755	0.740	0.519
NBIQA [91]	0.734	0.715	0.494	0.779	0.763	0.522
OG-IQA [61]	0.641	0.621	0.468	0.683	0.673	0.516
PIQE [16]	0.197	0.170	0.115	0.208	0.194	0.127
SSEQ [92]	0.607	0.583	0.427	0.661	0.650	0.480
FLG-IQA	**0.780**	**0.753**	**0.561**	**0.822**	**0.801**	**0.603**

**Table 10:** Results of the significance tests. Symbol 1(−1) denotes that the proposed *FLG-IQA* algorithm is significantly (95% confidence interval) better (worse) than the NR-IQA algorithm in the row on the IQA benchmark database in the column.

Method	CLIVE [45]	KonIQ-10k [46]	SPAQ [47]
BLIINDS-II [23]	1	1	1
BMPRI [12]	1	1	1
BRISQUE [9]	1	1	1
CurveletQA [10]	1	1	1
DIIVINE [25]	1	1	1
ENIQA [89]	1	1	1
GRAD-LOG-CP [11]	1	1	1
GWH-GLBP [90]	1	1	1
NBIQA [91]	1	1	1
OG-IQA [61]	1	1	1
PIQE [16]	1	1	1
SSEQ [92]	1	1	1

**Table 11:** Results of the cross-database test. The examined and the proposed methods were trained on KonIQ-10k [46] and tested on CLIVE [45]. The best results are in bold and the second-best results are underlined.

Method	PLCC	SROCC	KROCC
BLIINDS-II [23]	0.107	0.090	0.063
BMPRI [12]	0.453	0.389	0.298
BRISQUE [9]	0.509	0.460	0.310
CurveletQA [10]	0.496	0.505	0.347
DIIVINE [25]	0.479	0.434	0.299
ENIQA [89]	0.428	0.386	0.272
GRAD-LOG-CP [11]	0.427	0.384	0.261
GWH-GLBP [90]	0.480	0.479	0.328
NBIQA [91]	0.503	0.509	0.284
OG-IQA [61]	0.442	0.427	0.289
SSEQ [92]	0.270	0.256	0.170
FLG-IQA	**0.613**	**0.571**	**0.399**

## Data Availability

In this paper, the following publicly available benchmark databases were used: 1. CLIVE: https://live.ece.utexas.edu/research/ChallengeDB/index.html (accessed on 16 April 2022); 2. KonIQ-10k: http://database.mmsp-kn.de/koniq-10k-database.html (accessed on 16 April 2022); 3. SPAQ: https://github.com/h4nwei/SPAQ (accessed on 16 April 2022). The source code of the proposed FLG-IQA method is available at: https://github.com/Skythianos/FLG-IQA (accessed on 12 June 2022).

## Abbreviations

The following abbreviations are used in this manuscript:

BRIEF	binary robust independent elementary features
BRISK	binary robust invariant scalable keypoints
CGM	color gradient magnitude
CNN	convolutional neural network
CPU	central processing unit
DCF	dark channel feature
DCT	discrete cosine transform
DF	decision fusion
DSC	digital still camera
DSLR	digital single-lens reflex camera
FAST	features from accelerated segment test
FR	full-reference
GGD	generalized Gaussian distribution
GL	Grünwald–Letnikov
GM	gradient magnitude
GPU	graphics processing unit
GPR	Gaussian process regression
IQA	image quality assessment
KROCC	Kendall’s rank-order correlation coefficient
MOS	mean opinion score
MSCN	mean subtracted contrast normalized
NIQE	naturalness image quality evaluator
NR	no-reference
NSS	natural scene statistics
ORB	oriented FAST and rotated BRIEF
PIQE	perception-based image quality evaluator
PLCC	Pearson’s linear correlation coefficient
RBF	radial basis function
RM	relative gradient magnitude
RMS	root mean square
RO	relative gradient orientation
RR	reduced-reference
SPHN	smartphone
SROCC	Spearman’s rank-order correlation coefficient
SVR	support vector regressor
